# Recent Advances in Our Understanding of the Infectious Entry Pathway of Human Papillomavirus Type 16

**DOI:** 10.3390/microorganisms9102076

**Published:** 2021-10-01

**Authors:** Timothy R. Keiffer, Sarah Soorya, Martin J. Sapp

**Affiliations:** Department of Microbiology and Immunology, Center for Molecular and Tumor Virology, Feist-Weiller Cancer Center, Louisiana State University Health Sciences Center, Shreveport, LA 71130, USA; sarah.soorya@lsuhs.edu (S.S.); martin.sapp@lsuhs.edu (M.J.S.)

**Keywords:** HPV16, L1, L2, microtubules, vesicle, dynein, mitosis, PML

## Abstract

Papillomaviruses are a diverse viral species, but several types such as HPV16 are given special attention due to their contribution towards the pathogenesis of several major cancers. In this review, we will summarize how the knowledge of HPV16 entry has expanded since the last comprehensive HPV16 entry review our lab published in 2017.

## 1. Introduction

In this review, we will focus on the infectious entry of human papillomavirus (HPV), especially HPV type 16 (HPV16), the most intensely studied member of this group of viruses. We published a comprehensive HPV entry review in 2017 [1] and a review on the contribution of promyelocytic leukemia nuclear bodies (PML-NBs) in aiding HPV infection when the HPV genome enters the nucleus [2]. This review will expand on the knowledge of HPV entry discovered since our last entry review as well as highlight several directions that HPV entry research should go next and where our particular research will be focused on.

## 2. Structural Characteristics of HPV Capsid

The HPV capsid is a 55–60 nm diameter T = 7 icosahedral capsid composed of 360 copies of the major capsid protein L1 that are assembled into 72 pentamers, also called “capsomeres” [3,4,5,6,7,8]. Sixty capsomeres are hexavalent whereas 12 are pentavalent, meaning that they have six and five nearest neighbors, respectively. Capsomeres are interconnected by the disordered and flexible C-termini of L1 molecules. Additional stability is provided by L1 forming intercapsomeric disulphide bonds amongst conserved cysteine residues [9,10,11,12]. HPV16 L1 and other species expressed by themselves can form virus-like particles (VLPs) [12,13,14,15,16]. The minor capsid protein, L2, appears to be mostly buried/hidden underneath the capsid surface, with only amino terminal residues exposed to the surface and some L2 protein density observed at the capsomere base, corresponding to a little over 10kDa of L2 mass [6,17]. The L2 protein has over a half-dozen defined binding sites for cellular factors, such as cyclophilin B, syntaxin 18, and sorting nexins 17 and 27 [18,19,20,21,22,23]. L2 also contains motifs such as a transmembrane (TM) domain and nuclear export and nuclear localization sequences that are useful for its contribution to intracellular trafficking [24,25,26]. To date, the definite location and orientation of L2 within the mature HPV capsid remains a mystery despite recent advances by cryo-EM structure analysis [27].

## 3. HPV Binding to HSPGs

Our lab along with others in the HPV field utilize “pseudoviruses” (PsVs) to study HPV entry process, as PsVs are regarded as generally following the same entry pathway as “authentic” HPV particles [28]. Initially, pseudovirions were generated using viral expression vectors or yeast to express capsid proteins in cells harboring circular marker plasmids [29,30]. This system has now been greatly improved by using codon optimized versions of L1 and L2 and 293TT packaging cells. Briefly, 293TT cells are transfected with a reporter plasmid vector along with vectors to express high-levels of codon-optimized HPV16 L1 and L2 proteins. Marker expression is used as a readout of successful pseudogenome delivery [9,31,32,33]. Our lab uses PsVs to successfully infect a transformed keratinocyte cell line (HaCaT cells). HeLa cells are also used in the literature with PsVs for entry experiments.

HPV entry into cells requires capsid binding to extracellular matrix (ECM) as the ECM contains substrates for capsid binding and thus HPV infection. While PsVs can also directly bind to the cell surfaces of transformed cells, they preferentially bind to ECM components. ECM resident receptors identified for HPV16 include heparan sulfate proteoglycans (HSPGs) [34] and laminin-332 (previous designation, “laminin-5”) [35,36]. While laminin-332 is nonessential and can be bypassed by direct binding to cell surface resident heparan sulfates (HS), HSPGs are essential for infectious uptake of HPV16. Three HS binding sites have been identified on the HPV16 capsid by X-ray structure analysis and confirmed by mutational analyses [37,38]. Positively charged amino acids involved in these three interactions are derived from more than one L1 molecule. HSPG binding requires the correct folding of L1 protein into capsomeres. The three binding sites seem to be sequentially engaged initiating specific conformational changes within the capsid that in turn lead to a cascade of enzymatic cleavages of both L1 and L2 [39]. The serine protease kallikrein-8 cleaves L1, which is needed to add accessibility of the “buried” L2 protein within the capsid [40,41]. Additional action of host enzyme peptidyl-prolyl cis-trans isomerase cyclophilin B (CyPB) is needed to expose the N-terminus of L2 within the capsid [18]. Now the N-terminus of L2 is exposed and susceptible to cleavage by the furin convertase at a highly conserved cleavage recognition site for furin (R-X-K/R-R) at residues 9–12 [42,43,44]; furin precleavage of L2 within the virion bypasses the need for cellular furin, and furin pre-cleaved PsVs are used for entry experiments [42,45]. Alpha-defensin 5 blocks PsV16 infection by binding to the virion and preventing furin from cleaving L2, also causing virions to be redirected to the lysosome and degraded [46,47,48].

## 4. HPV Internalization and Capsid Uncoating

Next, HPV virion transport along filopodia assists with virion transport to the cell body and its efficient uptake [49,50]. HPV virions then utilize a non-HSPG secondary receptor for entry as the various conformational changes and cleavages on the L1 and L2 proteins decrease the affinity of the HPV virion for the HSPGs [39,51]. This non-HSPG platform and/or complex remains to be positively identified, but there are several main candidates thought to be part of this entry complex or implicated in its function: integrins α6 and β4, annexin A2, epidermal growth factor receptor (EGFR), and tetraspanins CD9, CD63, and CD151 [40,52,53,54,55,56,57,58]. The tetraspanins can interact with each other and form tetraspanin-enriched microdomains (TEMs) that then make contacts with other signaling molecules and proteins [59] that could organize this secondary platform HPV utilizes for entry. Expanding upon this, peptides that are mimics of the tetraspanin extracellular loop or C-terminal peptides comprised of CD63 and CD151 inhibit HPV16 entry [60]. Annexin A2 and its A2/S100A10 heterotetramer (A2t) are needed for HPV entry into cells; A2t heterotetramer is not required for HPV attachment [57]. Residues 108–126 of L2 bind specifically to the S100A10 subunit of A2t. A current model for A2t contribution to HPV entry is that HPV binding initiates EGFR signaling, leading to the phosphorylation of the A2t complex and subsequent endocytosis of HPV via lipid microdomains [56,61]. To our knowledge, there is no information on any contributions of phospholipids towards this HPV binding-mediated EGFR signaling. The tetraspanin CD9 affects HPV infection in a roundabout way by reducing ERK signaling via modulating the protease A Disintegrin and Metalloprotease (ADAM)17 [62]. ADAM17 activity is required for this HPV-entry complex as it is needed to form an HPV16/CD151/EGFR platform, although ADAM17 has not been shown to mediate HPV binding to target cells [63,64]. Elements of the cytoskeletal network such as obscurin-like 1 (OBSL1) also co-localize with L2, L1, and CD151, and they are required for HPV internalization, although whether the OBSL1 interaction with HPV occurs on the plasma membrane or in the endosome needs to be elaborated [65]. Individual HPV virions can be endocytosed into target cells quickly, within 2 h, but bulk entry of the majority of HPV virions can take much longer, up to 20 h, demonstrating that HPV entry is overall asynchronous [66]. Furthermore, this endocytosis appears to be independent of clathrin or caveolin mechanisms, but an actin-dependent pathway that is similar to, yet distinct from micropinocytosis [54,66]. Entry experiments performed with furin-precleaved HPV16 PsVs have indicated furin-processing is a rate-limiting step for HPV uptake [45].

During the last few years, several groups have studied HPV entry by observing the effect on HPV infection in cells over-expressing trans-membrane (TM) proteins or in cells expressing inducible small, artificial proteins. These proteins are comprised of primarily hydrophobic amino acids and are small, less than 25 residues in length. They are called “traptamers”, short for “transmembrane protein aptamers” [67]. The use of traptamers in HPV entry experiments is fairly new, with current data supporting traptamers affecting HPV entry later than the internalization step. Overexpression of the 88-residue TM protein stannin reduces HPV infection in both HeLa and HaCaT cells, promoting capsid protein degradation [68].

After endocytic internalization, the compartment containing the HPV capsid fuses with an early endosome (EE) as shown by L1 colocalization with the early endosome 1 (EEA) marker. Ras-related in brain (Rab) GTPase 5 (Rab5) is needed for HPV infection as it colocalized with HPV16; Rab5 is needed for function of EEs [66,69]. It is thought that the maturation of this HPV-laden EE compartment to the late endosome (LE) and then to a multi-vesicular body (MVB) triggers an uncoating of HPV virions in preparation for further intracellular trafficking to the nucleus [70]. CCT chaperonin complex was recently found to be involved in the intracellular processing of HPV [71]. The CD63/Syntenin-1 complex, along with the ESCRT pathway protein ALG-2-Interacting Protein X (ALIX), plays a role in trafficking HPV-containing EEs to the MVB [72]. Another protein comprising the ESCRT machinery, catalytically active VPS4, was also found to be necessary for capsid disassembly; VPS4 also forms a complex with the major and minor capsid proteins of HPV16 [73]. There is a newly found phosphorylation site on L2 at residue 62 that, if mutated, results in more trypsin stable L2 and the virus appears deficient in uncoating [74]. The kinase Pyk2 plays a role in capsid uncoating, but it is also thought to delay maturation of the EE to a LE [75]. Acidification of the EE is used by the partially uncoated HPV virion to dissociate L1 from L2 and the HPV genome and thus it is required for HPV infection [76]. Dissociated L1 protein is trafficked to lysosomes, however some L1 remains associated with the L2 and HPV genome during its subsequent intracellular trafficking [77,78,79,80].

## 5. HPV L2 Protein Penetrates through Endosomal Membrane

At this stage, the late endosome containing the HPV virion must enter the retrograde pathway or it is degraded in the lysosome [81]. L2 protein has the propensity to insert into cellular membranes via a carboxyl terminal amphipathic domain in the absence of heat shock proteins [82,83]. DiGiuseppe et al. demonstrated in 2015 that L2 protein indeed penetrates the limiting endocytic membrane during infection resulting in a transmembrane configuration with only 45 amino terminal amino acids remaining luminal [84]. The basic component of the above-mentioned amphipathic peptide of HPV16 L2 protein (RKRRKR), residues 457–462, that resemble a cationic cell- penetrating peptide (CPP) [85] is required to translocate across the endosomal membrane and into the cytoplasm. The carboxyl terminus of L2 becomes sensitive to protease digestion and accessible to antibody binding in infected physically disrupted cells [84] and can be bound by cytosolic transport factors [22,23,86,87]. L2 transmembrane configuration requires acidification of the endocytic vesicle [76,84], partial uncoating of the capsid [76,82], and cyclophilin chaperone activity [19]. It was previously known that chemical inhibition of y-secretase complex inhibits HPV infection [88,89]. The HPV genome does not reach the trans-Golgi network (TGN) in y-secretase-inhibited cells [90]. The active y-secretase complex is made up of the nicastrin (Nic), anterior pharynx defective 1 (APH-1), presenilin 1 (PS1), and the presenilin enhancer 2 (PEN-2) proteins [91]. The connection between y-secretase and infectious HPV entry has been further elaborated on since our last review. First, the protein p120 is required for HPV to engage with y-secretase [92]. p120 has previously been shown to recruit cadherins to the y-secretase complex via PS1 binding to p120 [93]. While y-secretase cleaves L2 protein as the HPV virion is in the endosome, this cleavage is not sufficient for infection. Rather, the y-secretase complex appears to act as a chaperone to mediate L2 insertion through the endosome in a pH-dependent manner, where the C-terminus of L2 can now act as a platform for cellular factors to mediate trafficking [94,95]. Furthermore, this y-secretase-mediated L2 insertion through the endosome seems to be transient and requires binding to its retromer complex partners for stabilization [96].

## 6. HPV Genome Ensconced by Vesicular Structure

At this point in HPV entry, the available data support a model where the HPV genome is protected by an endosome-derived vesicular structure with L2 protein protruding from the vesicle and into the cytoplasm, where it can act as a substrate to facilitate intracellular trafficking using cellular factors [1,97]. An obvious benefit for HPV utilizing this vesicular structure is to sequester its genome away from cellular sensors such as the cGAS/STING pathway as a robust interferon response is not detected in HPV-infected cells [98]. While this novel method of genome shielding prevents innate immune signaling, pre-treatment of cells with interferon-γ still results in a marked decrease in HPV infection, with the HPV genome found to be sequestered in the late endosome [99]. These findings suggest that interferon-γ, if introduced prior to infection, can protect cells from becoming infected by HPV16.

## 7. HPV Genome Trafficking to the TGN and during Mitosis via L2

The L2 protein has been shown by pulldown experiments and infection experiments to interact directly with the VPS35 subunit of retromer complex [100], helping to confirm the results of a previous siRNA screen implicating retromer as an HPV entry factor [101]. The retromer is comprised of a core protein trimer made up of VPS26, VPS29, and VPS35 proteins that facilitates retrograde trafficking from the endosome to the TGN along with several sorting nexin (SNX) proteins such as SNX17 and SNX27 [23,81]. The L2 protein of HPV16 has two retromer-recognition motifs on its C-terminus which are exposed following endosome membrane penetration, “FYL”, residues 446–448, and “YYML”, residues 452–455. Mutating these residues in L2 results in genome accumulation in the EE [100]. Hi-jacking the retromer complex so the HPV-laden vesicle can escape degradation and traverse this retrograde pathway to the TGN also requires utilizing the correct SNXs along with Rab proteins such as Rab7b and Rab9a [102]. The retromer associated protein TBC1D5, upon retromer complex binding to L2 protein, then hydrolyzes GTP via Rab7 to place the HPV on the retrograde pathway [103]. SNX17 interacts with L2 early after infection, estimated about 2 to 9 h post-infection [22]. As stated before, retromer complex binding after L2 pierces through the membrane is required to stabilize the membrane insertion of L2 [96]. Thus, infection can be blocked at this step using cell-penetrating peptides derived from the CPP and a retromer binding moiety from L2 or by the aforementioned traptamers that interfere with retromer association with L2 [67,104]. Complicating this part of the trafficking picture a bit more is the recent finding that L2 interacts with both the retromer and retriever complexes. HPV16 L2 interacts with the C16orf62 subunit of the retriever complex in an analogous matter to L2 with VPS35 of the retromer complex [86,100].

The L2 with its HPV-containing vesicle is thought to be sorted to the TGN in part due to endosomal tubulation. HPV infection was found to stimulate endosomal tubulation and if this is blocked via loss of the vesicle-associated membrane protein (VAMP)-associated protein, (VAP), the result is an infection block prior to HPV genome trafficking to the TGN [105,106]. VAP contacts the endoplasmic reticulum, TGN, and endosomes, which allows for endosomal tubulation [70]. There is some evidence to suggest that the HPV genome does enter the ER, although this requires further investigation [90,107]. At this point, HPV genome-laden vesicles are at the TGN waiting for the initiation of mitosis [70].

**Figure 1:**
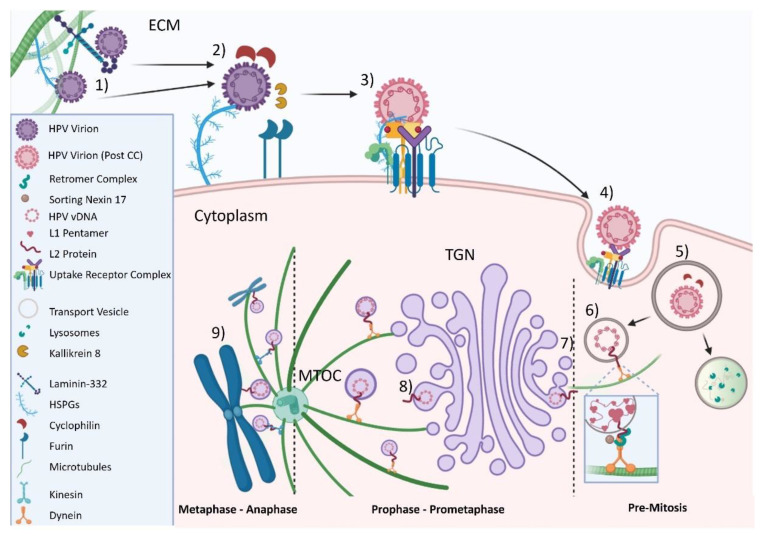
Schematic diagram of the late cytosolic trafficking events and nuclear delivery of incoming HPV16 genome. (**1**) Attachment of HPV virion to HSPGs on the extracellular matrix (ECM) with serine protease kallikrein 8 (KLK8) facilitating extracellular cleavage of L1. (**2**–**3**) Conformational changes in both L1 and L2 proteins. (**4**) Virions and uptake receptors internalized into cell. (**5**) Viral capsid uncoated; cyclophilin releases L1 and L2 afterwards. (**6**) L2 protein penetrates intercellular membrane and interacts with cytosolic factors. (**7**–**8**) Prophase-pro-metaphase: vesicles containing HPV genome dissociate from TGN and assemble on astral microtubules (MTs) to microtubule-organizing center (MTOC). (**9**) Metaphase -anaphase: vesicles assemble on spindle microtubules and travel to condensed chromosomes via (presumably) kinesin(s). Adapted from [1] and created with BioRender.

It is now well-established that HPV utilizes mitosis as part of its lifecycle and that nuclear envelope breakdown is another rate-limiting step for HPV infection [78,108,109]. The initiation of mitosis causes fragmentation of the TGN [110,111]. Using chemical agents to disburse the TGN does not prevent L2 and HPV genome trafficking to the nucleus [112], highlighting the importance of mitosis in HPV trafficking and infection. Knockdown of the enzyme dopachrome tautomerase (DC) reduces HPV infection of HaCaT cells by approximately 70%. Tracking EdU-labeled PsV16s in DC knock-out cells shows a retention of PsV16 genomes in ER, thought to be caused by DC knock-out disrupting cell-cycle progression and not interfering with HPV binding or internalization [80]. Upon initiation of mitosis, vesicles containing HPV genome along with L2 traffics along astral microtubules in a minus-end fashion towards the centrosome [78,112], presumably using at least the motor protein dynein [83,113]. More recent data from Lai et al. indicated that L2 forms a novel complex with the understudied Ran-binding protein 10 (RanBP10) and nuclear import protein karyopherin 2 (KPNA2) during trafficking. Furthermore, the role of dynein in infectious HPV trafficking was expanded upon as chemical inhibition of dynein decreased HPV genome import into the nucleus. HPV genome nuclear import was also decreased with knockdown of dynein light chain 3 (DYLN3) [87]. The dynein interaction with L2 has been mapped to residues 456–461 on L2 of HPV16 [83]. Indirect evidence suggests that plus end-directed transport along spindle microtubules is required as well, likely mediated by L2 protein [87]. Alanine substitution of arginine residues at position 302 and 305 results in loss of viral genome from spindle microtubules without affecting transport to the microtubule organizing center (MTOC) [79,84]. The same L2 domain may be responsible for tethering the transport vesicles to mitotic chromosomes [87]. The steps of HPV16 viral entry, from capsid binding to vesicle-protected genome transport via L2 to the condensed chromosomes, are pictorially summarized in Figure 1.

## 8. Nuclear Translocation and HPV Genome Association with Chromatin and PML NBs

After the HPV genome-harboring vesicle with L2, along with the aforementioned small amount of L1 associated with the HPV genome as capsomers, enters the nucleus, the HPV genome-laden vesicle remains intact until after mitosis is complete and the nuclear envelope is reformed [77,78,112,114]. The presence of membrane bound vesicles in the nucleus is challenging textbook knowledge, but HPV harboring vesicles have recently been visualized by electron microscopy [114]. It is at this stage of the HPV lifecycle where the HPV genome interacts with PML NBs.

PML NBs targeted by HPV have been proposed to have a protective role during its early viral cycle stages [2,115,116,117]. Specifically, proteins of PML NBs are utilized by HPV during the initial viral entry process for the efficient establishment and maintenance of the viral genome [2,78,84,115,116].

PML NBs are subnuclear punctate ring-like structures that are comprised of a few permanent and a large number of transient proteins, making PML NB composition incredibly versatile and dependent on cellular states [118,119]. The PML NB components implicated in being involved HPV infection are: PML protein, Sp100, DAXX, and (small ubiquitin-like modifier (SUMO) protein-1 (SUMO-1)) [116,117,120,121]. PML NB formation and recruitment of resident proteins is regulated by mainly SUMOylation, a vital post-translational protein modification. Many PML NB residing proteins harbor SUMO interacting motifs (SIMs) and SUMO conjugation motifs [122,123,124]. Interaction with SUMO through SIM regulate association as well as dissociation of proteins with PML NBs [125,126,127,128,129].

PML NBs become disorganized during mitosis, initiated by de-SUMOylation of PML protein [126,130]. PML protein molecules reassemble to form large aggregates referred to as mitotic accumulations of de-SUMOylated PML proteins (MAPPs) in the cytoplasm [131]. HPV does not associate with these MAPPs as they remain in the cytosol while the viral genome trans-locates to the nucleus [2,77,131] despite one report suggesting otherwise [132]. Different PML protein isoforms have been described with varying functions due to alternative splicing: isoforms I–VI reside in the nucleus, while isoform VII resides in the cytosol due to the lack of a nuclear localization signal [2,133].

After completion of mitosis and reformation of the nuclear envelope, PML proteins are associating with HPV-harboring nuclear vesicles before the HPV genome egresses from its vesicular structure [2,117]. It seems safe to speculate that L2 protein is involved in the recruitment of PMLs. Indeed, the carboxyl terminus of L2 protein from HPV33 has been shown to harbor a domain essential for L2 association with PML NBs in overexpression conditions [134]. SUMO can be detected as well at this stage, suggesting that PML protein is SUMOylated. In contrast, Sp100 is recruited to these sites only after viral genome has egressed from the membrane-bound vesicle [117].

Surprisingly, HPV genomes are lost after successful nuclear delivery if PML proteins are absent. This genome loss can be partially abated by chemically inhibiting the Jak/STAT pathway [116]. It is tempting to speculate about the existence of an innate immune sensor that is capable of sensing incoming HPV16 in the nucleus and target it for degradation. One possible candidate is IFI16, which is transiently residing in PML NBs and a known sensor of viral DNA [135,136]. However, direct experimental support for this speculation is still lacking.

**Figure 2:**
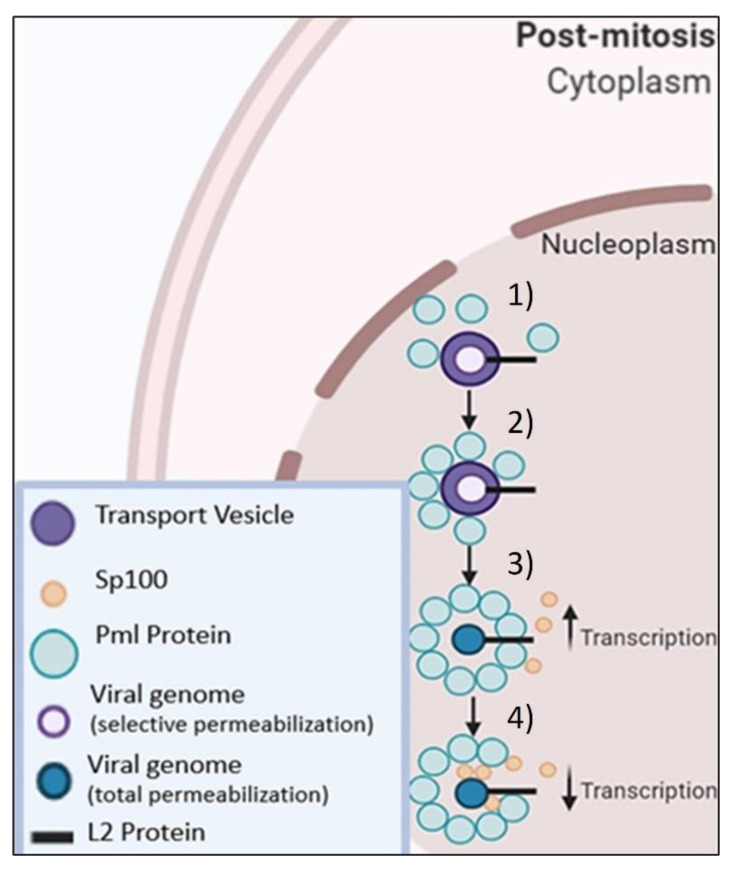
Working Hypothesis of PML & Sp100 Recruitment. (**1** & **2**) PML protein(s) are recruited and assembled around the vesicle. (**3**) Transcription rates increase with PML proteins surrounding vesicle. (**4**) Sp100 is recruited to the vesicles and decreases transcription of viral genome. Adapted from [2] and created with BioRender.

HPV16 L2 has a defined “chromosome binding region” (CBR) that was mapped to residues 188–334, and this CBR shares eight conserved residues between multiple HPV types, between residues 251–258, and more between residues 302–334 [137]. Mutations within or in close proximity to this CBR of L2 such as at residues: 286–289 (IVAL); 302/305 (R/R), and 313–315 (RTR) render the resulting virus unable to associate with chromatin [112,137]. L2 also has several SIMs, present at residues: 105–109, 145–148, and 286–289 [138,139]. If these SIMs on L2 are mutated, the resultant viruses have varying genome nuclear delivery deficiencies [117]. Our working hypothesis on the recruitment of PML protein(s) and Sp100 to the HPV genome and their subsequent effects of HPV16 genome transcription are summarized in Figure 2.

## 9. Concluding Remarks

The main advances in the study of HPV entry from our last review include further elaboration on the secondary receptor utilized by HPV as an entry platform(s), further confirmation of the existence of the vesicular structure used by the HPV genome during intracellular trafficking and nuclear import that we initially proposed, the identification of gamma-secretase as a chaperone required for L2 to engage retromer, and further elaboration of proteins involved in post-TGN HPV genome trafficking steps. Additionally, the DiMaio lab has started to use the aforementioned traptamer proteins to study HPV entry. These traptamers could be used to study other viral entry pathways.

There are at least two main avenues that warrant investigation about post-TGN HPV trafficking. First, further elaboration is needed on HPV trafficking from the TGN to the nucleus during mitosis. Lai et al. provide additional information on this that adds upon recent data from our lab and others [78,84,112] with their finding that L2, KPN2, RanBP10, and DYLN3 form a complex and mediate trafficking of HPV genomes via L2 and minus-end movement on astral (mitotic) microtubules toward the MTOC. However, there is no published data yet on the (proposed) plus-end movement from the MTOC to the condensed chromosomes. Approximately 50% of HPV genomes still travel to the nucleus in spite of siRNA-mediated knockdown(s) of RanBP10 and DYLN3 [87], showing that either there are still unknown adaptors that mediate minus-end movement via dynein or there is a dynein-independent pathway for HPV trafficking. We think the former is more likely. We are focusing on some candidate kinesin(s) potentially mediating this plus-end trafficking of HPV genomes via L2. Furthermore, only one kinesin was positively identified, Kif20b, by Lai et al. as a potential albeit weak L2 interactor in this system. The kinesin family is prodigious in size [140] and there are limited inhibitors available against kinesins. An alternative approach might be to utilize proximity ligation assays (PLAs) using antibodies against specific kinesins and EdU-labeled HPV pseudogenome(s) to determine if they interact. It may also warrant investigation into whether HPV utilizes the same adaptors/complexes in the different cell lines used to study HPV entry.

The second main avenue to follow is to determine how the HPV genome egresses from its protective vesicular structure to associate with the condensed chromosomes. One candidate facilitating this genome egress are phospholipases, as several of these have been identified by siRNA screens as being required for HPV infection [101,109]. This may also tie in with ascertaining the exact composition of the PML-NB complex that forms around the HPV-laden vesicle prior to genome egress. Virally encoded phospholipases are essential for endosomal egress in parvovirus infection [141], providing some precedence for this notion. However, HPV capsid proteins do not have signatures indicative of phospholipase activity. It will be interesting to test the hypothesis that HPV has usurped cellular lipases for egress. Indeed, several phospholipases are known to reside in the nucleus without a clear known function in that cellular compartment [142]. One phospholipase located in the nucleus, phospholipase C, gamma 1 isoform, has been shown to associate with PML-NBs [143], so there is a slight possibility that PML-NBs might recruit phospholipases to HPV genome-laden vesicles to facilitate viral DNA release. However, there is no additional data on nuclear phospholipase association or recruitment to PML-NBs to support this supposition.

Following up on HPV entry should continue to provide insights into how small tumor viruses evade the cellular immune response as well as identify new therapeutics and targets for HPV. There is an additional boon here with the use of traptamers for virology research and utilizing the CPP of L2 to deliver drugs or peptides into the cell.
